# Synthesis, Characterization, and Biological Evaluation of certain 6-methyl-2(3H)-benzo-1, 3-thiazolyl-1’-ethylidene-2-(o, p- Substituted Acetophenones) Hydrazine Analogs

**DOI:** 10.4103/0975-1483.71636

**Published:** 2010

**Authors:** G Alang, G Kaur, R Kaur, A Singh, R Tiwari

**Affiliations:** *G.H.G Khalsa College of Pharmacy, Gurusar Sadhar - 141 104, Punjab, India*; 1 *Department of Biotechnology, Punjab University, Chandigarh, India*

**Keywords:** Antimicrobial activity, substituted acetophenones, 2-aminobenzothiazole

## Abstract

In the present study, five new derivatives (GG4 to GG8) of benzothiazoles were synthesized and evaluated against *Staphylococcus aureus* (MTCC 737), *Pseudomonas aeruginosa* (MTCC 424), *Escherichia coli* (MTCC 1687), and yeast-like fungi *Candida tropicalis. p-Toluidine* on treatment with ammonium thiocynate formed 2-benzothiazolamines (II), which on reaction with hydrazine hydrate formed a hydrazino derivative (III). Compounds GG4 to GG8 were synthesized by reacting the hydrazine derivative with different acetophenones. All the synthesized compounds were identified by IR and ^1^H-NMR, and antimicrobial activity was performed on the synthesized compounds. Presence of NO_2_, Br, OCH_3_, and Cl groups to the substituted benzothiazole enhanced the antibacterial and antifungal activities.

## INTRODUCTION

**Figure 1:**
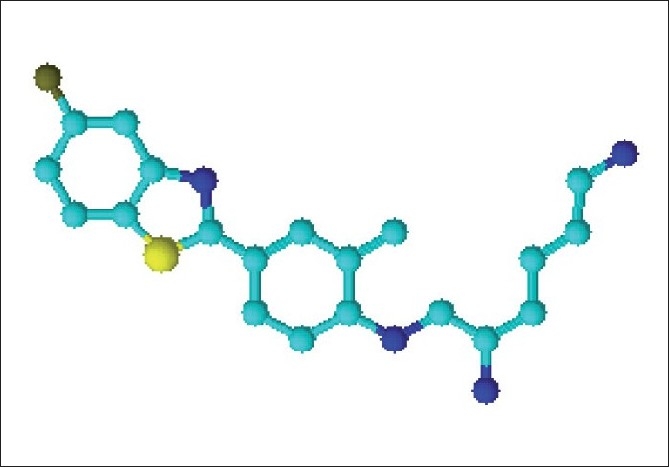
Molecular model of Phortress - An anti- tumor drug

’Riluzole’ the first benzothiazole containing an antiepileptic drug is the role model for the synthesis of various compounds with different activities based on a benzothiazole moiety. Since then, significant research has been carried out taking benzothiazole as the basic moiety. From the literature survey, it has been found that extensive work has been reported on 2-substituted benzothiazole derivatives in the past and evaluated for different activities, such as, antibacterial,[1] antiproliferative activity,[2] antiviral,[3] antitumor,[4] antifungal,[5] anti-inflammatory,[6] antioxidative and radioprotective,[7] antidiabetic,[8] antihelmintic,[9] antileishmanial,[10] anticonvulsant,[11] antimycobacterial,[12] neuroprotective,[13] and antipsychotic.[14] There are a number of pharmaceuticals and nutraceutical drugs available in the market containing benzothiazole moiety, reported to have different clinical uses. Phortress, [Figure 1] an antitumor drug has shown promising results in the clinical trials. Taking this into consideration, certain derivatives have been synthesized taking benzothiazole as the basic moiety.

### Experimental

All the chemicals and solvents used during the experimental studies were of analytical grade and were procured from CDH, New Delhi and Sigma Chemicals, Mumbai. Melting points of all synthesized compounds were determined by using an open capillary tube and were uncorrected. Infrared (IR) data were recorded in KBr disks, on a Perkin Elmer R-IX FTIR spectrophotometer, and the H^1^ NMR spectra on Bruker AC 30 of the NMR spectrometer 400 MHz.

## CHEMISTRY

### *p-Tolylthiourea* (I)

*p-Toluidine* (5.35 g) was dissolved in a mixture of concentrated HCl (4.3 ml) and water (11.6 ml) by heating in a water bath. The contents were cooled and solid ammonium thiocyanate (3.5 g) was added. The mixture was heated on water bath for about 22 hours. The precipitated product was cooled and filtered, washed with water three to four times, and dried. It was recrystallized with aqueous methanol to get cream colored crystals. Yield: 88% (m.p: 130°C). IR: 3435 (N-H*str*), 2999 (Aliphatic C-H*str*), 1612 (N-H*ben*), 1462 (Aromatic C=Cstr), 1310 (Aromatic C-H*ben*) ^1^H-NMR: 3.35 (2H, s, NH_2_), 7.24-7.11 (4H, d, Ar-H), 2.30 (3H, s, CH_3_).

### 2-Amino-6-methylbenzothiazole (II)

Fifteen milliliters of concentrated H_2_SO_4_ was added to p-tolylthiourea (8.3 g) and the temperature of the mixture was raised to 80°C on a water bath. Next, 48% HBr (0.5 g) acid was added slowly and the reaction mixture was stirred for two hours and set at 80°C. It was then cooled to room temperature and the reaction mixture was slowly introduced to cold water and then adjusted to pH 9 or 10 by adding ammonia water. The whole mixture was stirred for one hour by heating at 70°C and then cooled to room temperature. The mixture was extracted twice with dichloromethane and the combined extract was dried with anhydrous sodium sulfate and evaporated, to obtain the title compound. Yield: 80% (m.p: 145°C). IR: 3395 (N-H*str*), 3261 (N-H*str*), 1462 (Aromatic C=C*str*), 1326 (Aromatic C-N*str*), 1253 (C-S*str*). ^1^H-NMR: 3.45 (2H, s, NH_2_), 7.32-7.26 (3H, m, Ar-H), 2.34 (3H, s, CH _3_)

### 2-Hydrazino-6-methylbenzothiazole (III)

2-Amino-6-methybenzothiazole (20 g) [0.82 mmol] and hydrazine hydrate (85%) [0.11 mmol] in 50 ml of ethylene glycol were refluxed by stirring for four hours (60°C). The color of the reaction changed to green and a homogeneous solution appeared. A white solid was precipitated at the end of the reflux period. The mixture was cooled and the product was filtered and then washed with water several times. It was air dried and recrystallized by using ethanol. Yield: 43% (m.p: 192°C). IR: 3434 (NHNH*str*), 3162 (Aromatic C-H*str*), 3000 (Aliphatic C-H*str*), 1611.9 (N-H*ben*). ^1^H-NMR: 9.59 (1H, s, NH), 7.34-7.11 (5H, m, Ar-H), 3.37 (2H, s, NH_2_), 2.26 (3H, s, CH_3_).

### 2-{(3”-nitrophenyl)-1’-ethylidene}-hydrazinyl-6-methylbenzo-1, 3-thiazole (GG4)

2-Hydrazino-5-methylbenzothiazole (1.5 mmol), 3-nitroacetophenone (2.2 mmol), and glacial acetic acid (2–3 drops) were taken in absolute ethanol (20 ml) and refluxed on a water bath for eight hours, till different spots appeared, on *thin layer chromatography* (TLC). On cooling, the solid was separated. It was filtered and washed with little water and recrystallized with absolute ethanol. Yield: 48% (m.p: 181°C). IR: 3428 (N-H*str*), 3087.8 (Aromatic C-H*str*), 1613.9 (C=Nstr), 823 (Aromatic C-Nstr). ^1^H-NMR: 8.77 (1H, s, NH), 7.36-7.06 (7H, m, Ar-H), 2.38 (3H, s, CH_3_), 2.69 (3H, s, CH_3_).

### 2-{(4”-bromophenyl)-1’-ethylidene}-hydrazinyl-6-methylbenzo-1, 3-thiazole (GG5)

2-Hydrazino -5-methylbenzothiazole (1.5 mmol), 4-bromoacetophenone (2.2 mmol), and glacial acetic acid (2–3 drops) were taken in absolute ethanol (20 ml) and refluxed on a water bath for eight hours till different spots appeared, on TLC. On cooling, the solid was separated. It was filtered and washed with little water and recrystallized with absolute ethanol. Yield: 52% (m.p: 189°C). IR: 3434 (NH*str*), 3164 (Aromatic CH*str*), 1612 (C=N*str*), 1581 (NH*ben*), 699 (C-Br*str*). ^1^H-NMR: 9.58 (1H, s, NH), 7.26-7.12 (7H, m, Ar-H), 2.50 (3H, s, CH_3_), 2.27 (3H, s, CH_3_).

### 2-{(4”-methoxyphenyl)-1’-ethylidene}-hydrazinyl-6-methylbenzo-1, 3-thiazole (GG6)

2-Hydrazino-6-methylbenzothiazole (1.5 mmol), 4-Methoxyacetophenone (2.2 mmol), and glacial acetic acid (2–3 drops) were taken in absolute ethanol (20 ml) and refluxed on a water bath for eight hours till different spots appeared, on TLC. On cooling, the solid was separated. It was filtered and washed with little water and recrystallized with absolute ethanol. Yield: 41% (m.p: 169°C). IR: 3435 (N-H*str*), 3165 (Aromatic C-H*str*), 1612 (C=N*str*), 1581 (N-H*ben*), 1285 (Aromatic C-N*str*). ^1^H-NMR: 9.59 (1H, *s*, NH), 7.25-7.11 (7H, m, Ar-H), 6.72 (1H, s, NH_2_), 2.97 (3H, s, CH_3_), 2.33 (3H, s, CH_3_).

### 2-{(2”,4”-dichlorophenyl)-1’-ethylidene}-hydrazinyl-6-methylbenzo-1,3-thiazole(GG7)

2-Hydrazino-6-methylbenzothiazole (1.5 mmol), 2,4-Dichloroacetophenone (2.2 mmol), and glacial acetic acid (2–3 drops) were taken in absolute ethanol (20 ml) and refluxed on a water bath for eight hours till different spots appeared, on TLC. On cooling, the solid was separated, and was filtered and washed with little water and recrystallized with absolute ethanol. Yield: 54% (m.p: 177°C). IR: 3434 (N-H*str*), 3164 (Aromatic C-H*str*), 1612 (C=N*str*), 1582 (N-H*ben*), 800 (Aromatic C-Cl*str*). ^1^H-NMR: 9.59 (1H, s, NH), 7.25-7.11 (7H, m, Ar-H), 3.40 (3H, s, CH_3_), 2.26 (3H, s, CH_3_).

### 2-{(2”,4”-dimethoxyphenyl)-1’-ethylidene}-hydrazinyl-6-methylbenzo-1,3-thiazole(GG8)

2-Hydrazino-6-methylbenzothiazole (1.5 mmol), 2,4-Dimethoxyacetophenone (2.2 mmol), and glacial acetic acid (2–3 drops) were taken in absolute ethanol (20 ml) and refluxed on a water bath for eight hours, till different spots appeared, on TLC. On cooling, the solid was separated, and was filtered and washed with little water and recrystallized with absolute ethanol. Yield: 58% (m.p: 185°C). IR: 3433 (O-H and N-H*str*), 3163 (Aromatic C-H*str*), 1610 (C=N*str*), 1415 (Aromatic C=C*str*). ^1^H-NMR: 9.58 (1H, s, NH), 7.38-7.11 (7H, m, Ar-H), 3.38 (3H, s, OCH_3_) 2.26 (3H, s, CH_3_).

## RESULTS AND DISCUSSION

**Figure 2:**
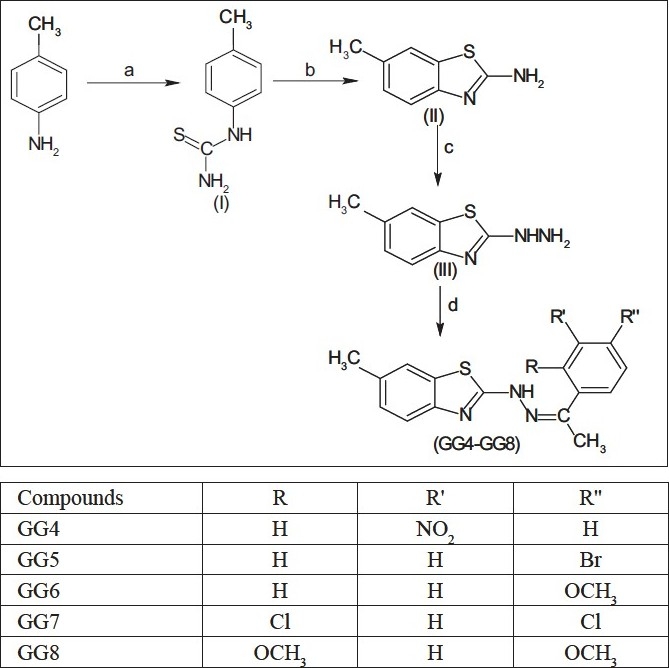
Reagents and Conditions; (a) ammonium thiocyanate, HCl, H^2^O, reflux, 22 hours; (b) HBr, H^2^SO^4^, reflux, 2 hours; (c) NHNH^2^, ethylene glycol, reflux, 4 hours; (d) appropriate substituted acetophenones, glacial CH^3^COOH, EtOH

The efficient synthetic route for the synthesis of benzothiazole derivatives is shown below [Figure 2]. *p*-Toluidine on reacting with ammonium thiocyanate formed p-Tolylthiourea (I), which on reaction with hydrobromic acid yielded 2-benzothiazolamines (II). This on reaction with hydrazine hydrate formed hydrazino derivatives (III). The compounds (GG4 to GG8) were synthesized by reacting with hydrazine derivatives, with different acetophenones (3-nitroacetophenone, 4-bromoacetophenone, 4-methoxyacetophenone, 2, 4-dichloroacetophenone, 2, 4-dimethoxyacetophenone).

### Antimicrobial activity

**Figure 3:**
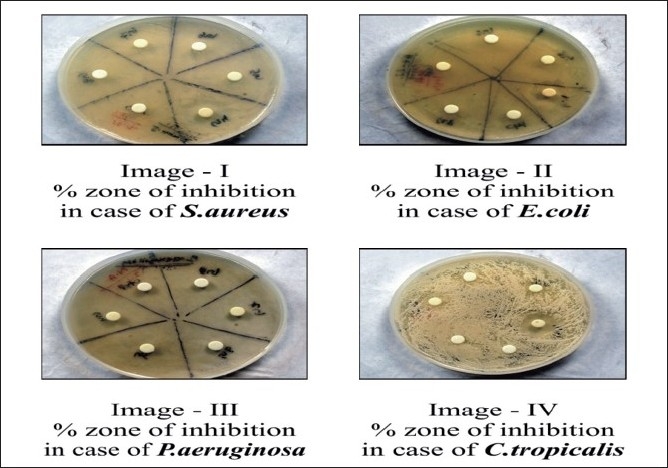
3% Zone of inhibition in different strains using the agar disk diffusion method

**Graph 1:**
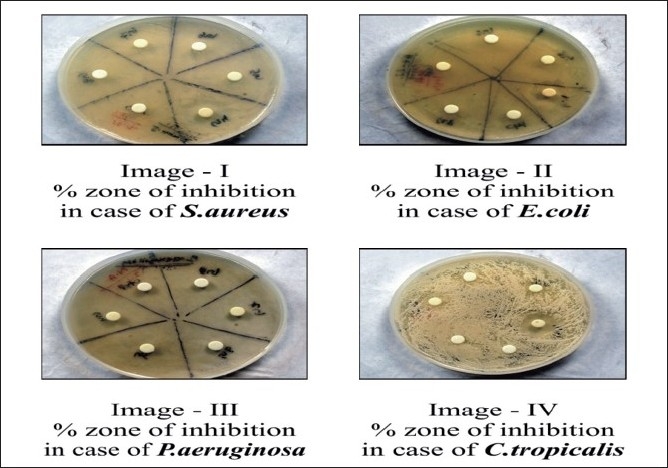
Comparison of % Zone of inhibition in case of *S. aureus*

**Graph 2:**
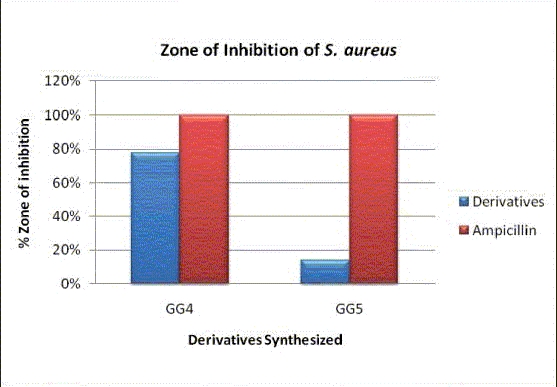
Comparison of % Zone of inhibition in case of *E. coli*

**Graph 3:**
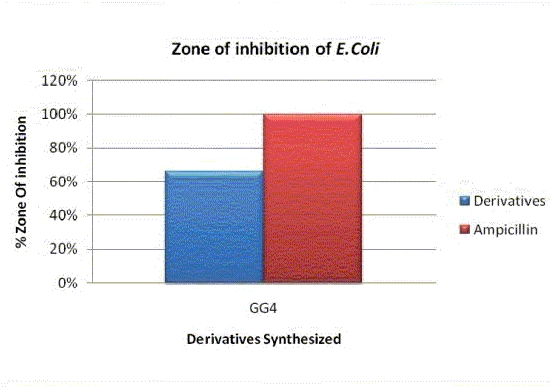
Comparison of % Zone of inhibition in case of *C. tropicalis*

In the present study, the efficacy of five new compounds was detected against **Gram positive bacteria — ** *Staphylococcus aureus* (MTCC 737), **Gram negative bacteria — ***Pseudomonas aeruginosa* (MTCC 424), *Escherichia coli* (MTCC 1687), and yeast-like fungi Candida *tropicalis*. The concentration of the test compound used was 50 mg/ml. Ampicillin and Clotrimazole were taken as the standard drugs [Tables 1 and 2]. Acetone was used as solvent control. The zone of inhibition obtained in different strains of bacteria and fungi are shown graphically in case of *S. aureus* [Graph 1], *P. aeruginosa, E. coli* [Graph 2], *C. tropicalis* [Graph 3], and with the help of original images taken [Figure 3], respectively.

**Table 1:** Comparison of the zone of inhibition of various synthesized compounds

Compounds	Anti-bacterial activity			Antifungal activity
	*S. aureus*	*E. coli*	*P aeruginosa*	*C. tropicalis*
Standard	14 mm	15 mm	14 mm	16 mm
GG4	11 mm (78)	10 mm (66)	–	11 mm (68)
GG5	2 mm (14)	–	–	5 mm (31)
GG6	–	–	–	2 mm (12)
GG7	–	–	–	6 mm (37)
GG8	–	–	–	–

Figures indicates in parentheses are in percentage

**Table 2:** Comparison of antimicrobial activity with different synthesized compounds

Compounds	Anti-bacterial activity			Antifungal activity
	*S. aureus*	*E. coli*	*P aeruginosa*	*C. tropicalis*
Standard	+++	–	+++	+++
GG4	+++	–	–	+++
GG5	+	–	–	++
GG6	–	–	–	+
GG7	–	–	–	++
GG8	–	–	–	–

+++ Diameter of zone of inhibition between 11 and 16 mm, ++ Diameter of zone of inhibition between 5 and 10 mm, + Diameter of zone of inhibition between 2 and 5 mm, - No zone of inhibition observed

Compound GG4 showed significant activity against *S. aureus, E. coli*, and *C. tropicalis* when tested at 50 mg/ml concentration taking ampicillin and clotrimazole as the standard. From the SAR studies, the presence of the electron withdrawing group (i.e., NO_2_) in compound GG4 was assumed to be responsible for the observed activity. From the above-mentioned results, it may be concluded that the derivatives of benzothiazoles possess moderate-to-potent antimicrobial activity[1,5] when compared to the standards. Furthermore, other sites (6 and 7) available at the benzothiazole moiety would be explored, in order to obtain compounds with different activity and potent antimicrobials. Therefore, the present study will help scientists in future, to undertake a different mode to synthesize more potent antimicrobials.
